# Financing child rights in Malawi

**DOI:** 10.1186/s12889-023-16319-x

**Published:** 2023-11-16

**Authors:** Rachel Etter-Phoya, Chisomo Manthalu, Frank Kalizinje, Farai Chigaru, Bernadetta Mazimbe, Ajib Phiri, Takondwa Chimowa, Waziona Ligomeka, Stephen Hall, Bernadette O’Hare

**Affiliations:** 1 Tax Justice Network, Lilongwe, Malawi; 2 University of St Andrews, St Andrews, UK; 3 Oxfam, Lilongwe, Malawi; 4 University of Pretoria, Pretoria, South Africa; 5 University of Malawi, Zomba, Malawi; 6 Sheffield Hallam University, Sheffield, UK; 7 Kamuzu University of Health Sciences, (KUHeS), Blantyre, Malawi; 8 Ministry of Health, Lilongwe, Malawi; 9 Zomba Central Hospital, Zomba, Malawi; 10 Ministry of Finance, Lilongwe, Malawi; 11 School of Business, University of Leicester, Leicester, UK

**Keywords:** Child rights, United Nations Convention on the Rights of the Child, Illicit financial flows, International corporate tax avoidance, Tax evasion, Water, Sanitation, Education, Child mortality

## Abstract

**Background:**

Nearly all countries have ratified the United Nations Convention on the Rights of the Child and, therefore, support children having access to their rights. However, only a small minority of children worldwide have access to their environmental, economic, and social rights. The most recent global effort to address these deficits came in 2015, when the United Nations General Assembly agreed to a plan for a fairer and more sustainable future by 2030 and outlined the Sustainable Development Goals (SDGs). One remediable cause is the lack of revenue in many countries, which affects all SDGs. However, illicit financial flows from low-income to high-income countries, including international tax abuse, continue unabated.

**Methods:**

Using the most recent estimates of tax abuse perpetuated by multinational companies and tax evasion through offshore wealth, and precise econometric modelling, we illustrate the potential regarding child rights (or progress towards the SDGs) if there was an increase in revenue equivalent to tax abuse in Malawi, a low-income country particularly vulnerable to climate change. The Government Revenue and Development Estimations model provides realistic estimates of government revenue changes in developmental outcomes. Using panel data on government revenue per capita, it models the impact of increased revenue on governance and SDG progress.

**Results:**

If cross-border tax abuse and tax evasion were curtailed, the equivalent increase in government revenue in one country, Malawi, would be associated with 12,000 and 20,000 people having access to basic water and sanitation respectively each year. Each year, an additional 5000 children would attend school, 150 additional children would survive, and 10 mothers would survive childbirth.

**Conclusions:**

More children would access their economic and social rights if actions were taken to close the gap in global governance regarding taxation. We discuss the responsibility of duty bearers, the need for a global body to arbitrate and monitor international tax matters, and how the Government of Malawi could take further domestic action to mitigate the gaps in global governance and protect itself against illicit financial flows, including tax abuse.

## Introduction

Children depend on critical services for their fundamental rights, including water, sanitation, and education. However, there is vast inequality in the coverage of essential services for children; while coverage approaches 100% in higher-income countries, it is much lower in lower-income countries.^1^ For example, in 2020, 25% of children did not drink safe water globally, and the figure was 70% in sub-Saharan Africa [1]. Climate change is amplifying these current environmental risks, and pre-existing deprivation reduces resilience to further shocks [2]. Insufficient government revenue is a major reason why children’s needs and rights are not met [3]. To illustrate the international community’s opportunity to contribute to progress on the Sustainable Development Goals (SDGs), and thus child rights in other countries, we use estimates of cross-border corporate tax abuse and tax evasion in one country, Malawi, and use the Government Revenue and Development Estimations models to show the potential in terms of child rights if the government had additional revenue equivalent to these losses. We discuss actions which the international community and the government of Malawi could consider to ensure more children in Malawi access their rights.

## Background

To address the deficits in the realisation of rights, the United Nations (UN) General Assembly agreed on a plan for a fairer and more sustainable future by 2030 and outlined the SDGs with associated targets and indicators. The SDGs are grounded in international human rights law, and here, we use the terms SDG progress and child rights interchangeably [4]. Governments agree that sustainable financing using domestic revenue is required to achieve the SDG agenda. However, SDG progress is off-track, in part, because of constrained government finances [5], and many countries, especially in sub-Saharan Africa, will not reach SDG targets by 2030 [6], partly because of these financing constraints.

### Government revenue, child rights, governance, and the Sustainable Development Goals

Reducing losses from public finances leads to increased spending across public services in multiple sectors, such as education, agriculture, and infrastructure, which impacts children’s rights and drives SDG progress [7–11]. Tax is the primary means of implementing the SDGs, as most government income is derived from taxation [12]. However, tax gaps—the difference between actual collection and potential—mean that revenue is insufficient to achieve child rights. This holds particular salience for lower-income countries, whose spending on social sectors, as a proportion of the budget, is larger than that in higher-income countries [13, 14], and the gains in terms of SDG progress from marginal increases in revenue would be more substantial [15]. Thus, small revenue increases result in significant increases in coverage of SDG indicators, and thus it is critical to tackle tax-motivated illicit financial flows.

Tax gaps are comprised of both domestic and international components. Domestic tax gaps could be reduced by increasing domestic resource mobilisation, and international tax gaps could be reduced by curtailing global losses [16]. Corporate tax from multinational companies is an essential source of government revenue, especially in lower-income countries [17]. The international tax gap is mainly due to corporate tax evasion and avoidance, and in the context of a narrow tax base, curbing this is the most feasible way to fund the SDGs in the short term [18, 19]. Thus, curtailing tax gaps is essential to increasing fiscal space, which would reduce the use of regressive tax policies to raise revenue and reduce debt accumulation to cover shortfalls in national budgets [20].

Alongside revenue, governance impacts SDG progress, and government spending achieves better results in well-governed countries [21, 22]. Well-governed countries are more likely to encourage business sector development and investment, enjoy economic growth [23, 24], and have robust institutions and political stability [25, 26]. Thus, the impact of any additional government revenue is significantly amplified in well-governed countries, especially when revenue is scarce [27]. Indeed, there is a two-way relationship between government revenue and governance, which drives a virtuous circle and improves the generation of further revenue and the allocation and efficient use of additional resources [15].

### The risk of illicit financial flows

The ability of governments to finance development is undermined by illicit financial flows that cause revenue losses. Illicit financial flows—“financial flows that are illicit in origin, transfer, or use, and that cross country borders”—are facilitated by jurisdictions that offer secrecy or lower or zero tax rates to residents and companies operating in other countries [23]. This problem is acknowledged in the SDG agenda. For example, target 16.4 of the 16^th^ SDG aims to reduce illicit financial flows significantly [28]. This is the first globally agreed target to curb illicit financial flows, acknowledging the cross-border impact of national tax and financial policies and laws.

The importance of tackling illicit financial flows to increase the fiscal space for self-financed sustainable development was elevated following the establishment of the High Level Panel on Illicit Financial Flows from Africa, which was mandated by the African Union Commission and the UN Economic Commission for Africa Conference of African Ministers of Finance, Planning, and Economic Development in 2011 and chaired by Thabo Mbeki, former South African president. This informed the development of the SDGs as well as the UN’s adoption of the Addis Ababa Action Agenda, including commitments to address illicit financial flows as part of financing development [29]. According to the High Level Panel’s landmark report, often referred to as the Mbeki Report, published by the panel in 2015, commercial practices, including tax abuse, are the primary drivers of illicit financial flows from Africa, followed by criminal activities and corruption [23]. The four types of illicit financial flows can be distinguished from each other based on motivation and include 1) market/regulatory abuse, 2) tax abuse, 3) abuse of power, including theft of state funds and assets, and 4) proceeds of crime [12]. These flows sit on a spectrum of legality; some are clearly illegal, such as laundering the proceeds of crime and theft of state funds, while other transactions may be considered legal, such as instances of tax avoidance, at least when they have not been challenged in court [12]. The commercial category, or tax-motivated illicit financial flows, reduces government revenue and resources for public services. These include tax avoidance and evasion. Tax avoidance is the practice of minimising tax bills by taking advantage of loopholes or interpreting a tax code in an unintended way. In contrast, tax evasion intentionally defrauds revenue authorities. Both have the same harmful effects on government revenue.

Annual tax losses have been estimated at US$483bn worldwide due to cross-border tax abuse by multinational companies and offshore tax evasion [30]. The vulnerabilities that enable these tax losses are primarily created by member countries of the Organisation for Economic Co-operation and Development (OECD), some of which are former imperial powers and the wealthiest nations in the world, all of whom, with the exception of the USA, have ratified the United Nations Convention on the Rights of the Child [31]. Specific economic channels—exports and imports, inward and outward foreign direct investment, portfolio assets and liabilities, and banking claims and liabilities—and particular bilateral relationships between countries, especially with tax havens, increase the vulnerability and exposure of countries to illicit financial flows [32]. In Africa, Abugre et al. found that European dependent jurisdictions, and especially the UK’s crown dependencies and overseas territories, provide a disproportional share of risks of illicit financial flows in Africa across the economic channels [32].

### Tax abuse and child rights

National and international obligations for children’s economic and social rights (child rights), including rights to clean air, safe water, sanitation, nutritious food, and education, are enshrined in the most widely ratified human rights instrument, the United Nations Convention on the Rights of the Child (UNCRC). All countries that have ratified the UNCRC have responsibilities for children in other countries alongside their domestic obligations. This responsibility includes promoting international cooperation to realise children’s rights everywhere and accounting for the needs of lower-income countries [33]. Although countries are generally considered primarily responsible for respecting, protecting, and fulfilling their children’s rights, they are often hampered in their efforts to meet these obligations and raise revenue to finance rights, in part because of the dynamics of international finance, trade and tax. The UN Committee on Economic Social and Cultural Rights’ General Comment Number 24 clarifies that states are responsible for the action of companies, including banks domiciled on their territory [34]:

States Parties are required to take the necessary steps to prevent human rights violations abroad by corporations domiciled in their […] jurisdiction […] without infringing the sovereignty or diminishing the obligations of the host States under the Covenant.

Here, international human rights law assigns duties to states, not companies, and international tribunals rarely extend jurisdiction over legal persons, including companies.

Since at least 2014, UN human rights experts and committees have made comments on the implications of cross-border tax abuses on human rights [35]. For example, in 2020, in their concluding remarks on the review of Ireland, the UN Committee on the Rights of the Child [36] demonstrates the connection between domestic tax policy and the impact globally on child rights,

Ensure that tax policies do not contribute to tax abuse by companies operating in other countries, leading to a negative impact on the availability of resources for realising children’s rights in those countries. (para 10c)

This is the first time that the UN Committee on the Rights of the Child has considered the effect of a country’s tax policies on children’s rights overseas. In 2022, the committee made similar concluding remarks on the review of the Netherlands [37]. Thus, international human rights law has specifically established that jurisdictions have extra-territorial responsibility for the impact of their rules and legislation that enable cross-border tax abuse because they undermine fundamental rights, including women’s and children’s rights.

### Child rights in Malawi

Malawi has made considerable progress in child rights, and since 2000, under-five mortality has plummeted from 172 per 1000 live births in 2000 to 39 per 1000 live births in 2020 [38]. However, there are huge gaps in children’s access to fundamental rights. For example, one-quarter of Malawian children do not have access to basic water, half do not access basic sanitation, and one-third are stunted, indicating chronic malnutrition [39]. Although 90% of children enrol in primary school, only one-third complete it [39]. On an index of SDG achievement, which measures progress towards all 17 goals as a percentage, Malawi scores 53% and ranks 145^th^ out of 163 countries in 2022 [40]. There is a high risk that the impacts of climate change will slow SDG progress, and in particular affect children in Malawi, where Malawian children are among children from 40 countries most vulnerable, according to UNICEF’s Children’s Climate Risk Index [2]. At the population level, an increase in revenue has been shown to increase government expenditure on public services, and as a result, it will have a positive impact on health determinants and educational outcomes [31]. Of course, at the individual and household levels, there are many different factors that determine health and educational outcomes, including socio-economic circumstances and inequities [28, 41, 42]. 

### Public finance and illicit financial flows in Malawi

The fiscal space in Malawi is limited. Government revenue as a percentage of Gross Domestic Product (GDP) is 12.6% (excluding grants) [43]. GDP per capita is $394 2015 USD [38], therefore, government revenue per capita was $50 2015 USD in 2020 [44]. Until recently, donors had contributed 40% to the national budget in Malawi (called budget support), but some donors halted this in 2013 due to the misuse of public finances, and budget support decreased to 15%. Donors redirected their support to programmes (especially in education and health) outside the budget, but this support has been criticised for poor coordination and duplication [45, 46]. Given the reorientation of official development assistance away from budget support, the government assumes that the country’s development will depend mainly on domestic resources [47], and self-reliance is the longer-term policy aim as outlined in Malawi’s Vision 2063 [48]. The government has relied on borrowing to compensate for fiscal imbalances resulting from the reduction in aid. Consequently, Malawian debt increased from 30% to 50% of GDP between 2011 and 2020, of which 20% is non-concessional external debt, and it is projected to increase to 85.7% of GDP in 2026 [49, 50]. Unless restructuring occurs, about one-third of the government’s revenue (excluding grants) will be allocated to servicing debt over the next few years [51], adversely affecting revenue allocation to child rights and all the SDGs.

The Government of Malawi’s policies to increase public finances and effectiveness include tackling different forms of illicit financial flows, driven by commercial, criminal and corrupt practices. The Malawi Revenue Authority singles out the prevention of tax avoidance and tax evasion in its Domestic Revenue Mobilisation Strategy 2021–2026 [52] and the Financial Intelligence Authority’s most recent national assessment demonstrates action on the three drivers of illicit financial flows [53].

In 2022, the Government of Malawi through the Ministry of Finance along with the African Union Commission and the Coalition for Dialogue on Africa assessed Malawi’s progress on implementing the recommendations of the aforementioned Mbeki Report. It notes that Malawi has a fairly robust legal and regulatory framework. However, effective implementation is curtailed by inadequate resources, weak coordination between institutions and political interference. The lack of cooperation from partner countries is a further limitation [54]. UNCTAD is currently piloting six methodologies to support the 2030 Agenda for Sustainable Development by defining, measuring and disseminating statistics on illicit financial flows; Malawi is not yet among the African countries where methodologies are being piloted [55]. The Illicit Financial Flows Vulnerability tracker, published by the Tax Justice Network, based on the work of Abugre et al. in 2019 [56] and building on analysis in the Mbeki Report, demonstrates the channels where Malawi is most vulnerable to illicit financial flows; on average, between 2009 and 2019, these include inward and outward foreign direct investments alongside inward portfolio investment and imports, which suggest that Malawi is at risk of tax avoidance, trade-based money laundering, and tax evasion, although it is not possible to determine the specific types of illicit financial flows without transactional level data [57].

Based on the cases investigated and/or prosecuted between 2013 and 2017, the Financial Intelligence Authority identified just over US$17 million as the total amount of corruption-related proceeds [53], which includes prosecution following a period of large-scale theft of public money popularly known as Cashgate, “although the number of identified and investigated money laundering cases may not give an accurate and full-scale picture of the threat”. Their estimates for the same 5-year time period including undetected and unrecorded proceeds from criminal offences were as follows: US$46 million for illegal possession of protected species, US$30.5 million for illegal logging, US$27.6 million for illegal externalisation of forex, US$20.9 million for tax evasion, US$18.7 million for corruption, and US$1.5 million for smuggling [53]. These amounts averaged out on a yearly basis are smaller than estimates of tax abuse, and they form just one part of Malawi’s entire burden of illicit financial flows.

There are a few case studies, generally commissioned by non-governmental organisations, that seek to quantify the revenue losses associated with multinational corporations with subsidiaries in Malawi. Most studies have focused on the tax practices of Paladin Energy which until 2020 held a mining licence for Malawi’s largest mine, Kayelekera Uranium Mine, through majority shareholding in Malawian subsidiary Paladin Africa Ltd. It was the largest single foreign direct investment at the time. In a 2015 study by ActionAid International, the organisation stated that across 6 years Malawi lost US$43 million as a result of tax breaks awarded in the mining development agreement (US$15.63 million) and of lower withholding taxes owed in Malawi through the company’﻿s use of double tax treaties (US$27.5 million) [58]. Etter-Phoya and Malunga’s 2016 economic model of the mine concurred with foregone revenue totalling US$15 million as a result of tax incentives across five years of production [59]. Earlier studies exist, but they do not make their financial modelling explicit and made assumptions before production was suspended, such as one that estimated total annual losses of US$15 million [60]. Other multinationals, such as Malawi Mangoes, have come under media scrutiny for their tax arrangements and the potential impacts on tax revenue in Malawi [61]. However, the Malawi Revenue Authority does not systematically publish data on tax avoidance and evasion cases investigated and successfully concluded [62].

Inevitably, given their opacity, quantifying the scale of illict financial flows in any country, including Malawi, is complicated. The best data available is for tax-motivated illicit financial flows, which, according to the Mbeki Report, form the bulk of illicit financial flows, although there is variation from country to country. For these reasons, and given the cross-border obligations under international human rights law regarding tax policy, data on tax losses is the focus of the present study.

To illustrate the international community’s opportunity to contribute to SDG progress, and child rights in other countries, we use estimates of cross-border tax abuse, based on corporate profit shifting and offshore wealth. We previously presented the impact of tax abuse on SDG progress in multiple countries [31]. Here, we focus on both corporate and individual tax abuse in one country to allow us to consider domestic actions which this country (and others also) could consider to protect children from the impact of cross-border tax abuse.

We present current estimates for cross-border corporate and individual tax abuse in Malawi and the potential for increased government revenue equivalent to lost revenue in terms of SDG progress. We focus on Malawi because Malawi’s SDG progress is uneven and it was the southern African country most impacted by the war in Ukraine, with respect to its economic growth. Malawi's revenue as a percentage of GDP declined between 2019/2020 and 2020/2021 and curtailing illicit financial flows would contribute to SDG progress by increasing government revenue [63]. Progress is further hampered by climate change [2], debt distress [64], and dwindling overseas development assistance [65], which underlines the importance of capturing lost revenue. Further, additional revenue in lower-income countries, such as Malawi, has significant impact on SDG progress. Finally, and most importantly, the authors are familiar with the domestic policy context for both child rights and illicit financial flows.

## Methods

### Estimates for the international tax gap in Malawi

There is no consensus on the definition or methodology for quantifying illicit financial flows, even though there is now an agreement (target 16.4) in the SDGs to reduce illicit financial flows [12]. Therefore, estimates measure various dimensions of illicit financial flows, and there are several different methods of estimating tax abuse [17, 30, 66, 67]. We draw on a recent book by Cobham and Janský that provides the most comprehensive assessment of methodologies to estimate illicit financial flows as required to monitor progress on SDG target 16.4 implementation, culminating in two proposed indicators—one on tax avoidance by multinationals and the other on undeclared offshore wealth. Therefore, for estimates on the international tax gap in Malawi, we sought to estimate tax revenue losses associated with both corporate profit shifting and individual offshore wealth. O’Hare et al.’s study in 2022 uses estimates of tax abuse from the Global Alliance for Tax Justice, Public Services International and the Tax Justice Network’s State of Tax Justice 2020 [67]. We use Alstadsæter, Johannesen and Zucman’s hidden wealth [68, 69] and Tørsløv and Zucman’s missing profits [70] estimates. Tørsløv and Zucman’s estimates for missing profit are lower than other studies, including the State of Tax Justice, but both estimates are within the same range, as the Tax Justice Network’s methodological note points out [71]. Despite their limitations, we concur with Cobham and Jansky (2017), who consider these estimates to be, in part, the expert consensus in the sense that they reflect the authors’ informed perspective on how large the scale of the illicit financial flows problem is. Therefore, we interpret estimates loosely, meaning that they are in the range that experts expect them to be.

To estimate offshore wealth—i.e. the amount of foreign wealth managed by the banks of a jurisdiction—is difficult because of a paucity of statistics. The newly disclosed Bank of International Settlement data (BIS) on bilateral banking statistics on foreign-owned deposits in a number of tax havens is the best available data in a context where Switzerland is the only tax haven that publishes comprehensive public data, and even this does not always include the beneficial owners and thus may disguise the country of origin [12]. Alstadsæter, Johannesen and Zucman, building on Zucman’s earlier study that employed the Swiss central bank’s data [72], additionally use the BIS data, excluding interbank deposits that do not involve households, and IMF balance of payments and investment position data to allocate the amount of foreign wealth held in tax havens in 2007, prior to the widespread use of shell companies, which makes it difficult to identify the beneficial owner and therefore the country of nationality, origin or residence of asset and wealth holders. In their assessment, Cobham and Janský posit that this is likely the most reliable estimates of wealth in tax havens, yet with improved data, estimates will be more refined. For example, this does not include non-financial wealth, such as real estate [12]. Based on the country-level estimates of offshore wealth, we assumed a 5% return on this wealth and used the highest personal income tax band for Malawi (35%) to derive the country’s annual foregone tax revenue. We expressed it as a percentage of government revenue in 2007.

To estimate tax revenue losses from corporate profit shifting, the estimates from missing profits are used [73]. Tørsløv and Zucman calculate the excess profitability of foreign firms to local firms in tax havens using foreign affiliates statistics and national accounts data (which cover all firms—foreign plus local—incorporated in each country). Then the bounds for the probability of the differences between foreign and local firms’ profits due to true economic differences were provided. Then using new bilateral balance of payments data, the shifted profits are reallocated to the countries where the profits have been made. This is done by following where intragroup interest payments from tax havens are made and the destination of exports which are conducive to tax avoidance, such as the right to use intellectual property and management advice [73]. For Malawi, there is no data available and we assume, guided by the authors of missing profits [70], that 10% of corporate tax is lost due to corporate tax avoidance.

### Estimating the impact of revenue increases on SDG progress

We employ the economic modelling from the Government Revenue and Development Estimations project (the GRADE) [44] to translate the impact of an increase in revenue equivalent to the international tax gap on the number of children who could access their fundamental rights. The GRADE uses panel data to model the impact of increased government revenue on the SDGs [15, 74, 75]. If government revenue changes, the model shows the change in coverage of water, sanitation, child school years and survival as a percent. We use the UNU-WIDER Government Revenue Dataset to establish annual government revenue [76]. We then use population data (World Development Indicators, WDI) from each country and for each year to convert the change in percentage coverage into the number of people who will have access to these SDG indicators and the number of children and mothers who survive. The model incorporates the effect of additional revenue on governance indicators (using the Worldwide Governance Indicators, WGI) [77]. Additional revenue equivalent to tax losses drives SDG progress and directly improves governance, which in turn amplifies the impact of the additional revenue on SDG progress, leading to the generation of further revenue and thus a “virtuous circle” [78]. The GRADE modelling assumes that government spending of additional revenue mirrors recent government spending patterns and that an increase in revenue results in improvements for all SDGs and everyone’s rights. Because the modelling is directly between government revenue, governance and SDG indicator outcome, we do not assume that there is any changes to allocation decisions. This is more realistic than assuming additional revenue will be spent on any one sector. Further, the benefits of an increase take time to show, so the GRADE model estimates incremental benefits from increased spending.

Prior to the GRADE modelling, some studies unrealistically equated the total of tax losses directly to impact in a specific sector or budget line. For example, by calculating the number of public sector salaries equivalent to revenue foregone from a given mining company’s tax incentives and profit shifting, as in the aforementioned ActionAid International study in Malawi [58]. Limitations of the GRADE modelling include the absence of government revenue data prior to 1980, and data on governance quality from the WGI was unavailable before 1996. Data on maternal mortality is only available until 2017.

This paper focuses on the potential improvements for child rights, where clean water, sanitation, education and healthcare are among the most critical determinants of child health [79]. Therefore, we focus on SDGs 3, 4, and 6 [80]. The estimates provide point estimates of revenue losses, and we assume these estimates, as a percentage of government revenue, are constant over time, covering the time to be modelled. We present estimates for all years where there is data (from 2002 to 2020) because these losses occur over decades, and this is the maximum period where data is available. GRADE models the positive impact of an increase in revenue on governance and SDG progress, thus, the long-term virtuous circle this generates [81].

## Results

### Estimates of the cost of tax avoidance and evasion in Malawi

Missing profits estimates that as of 2019 countries like Malawi without country level data lose 10% of their corporate tax revenue due to tax avoidance [70]. Corporate income tax in Malawi is 2.3% of GDP [76]. We then express the losses in terms of government revenue, using the UNU-WIDER Government Revenue Dataset. In Malawi, the losses are equivalent to 1.85% of government revenue, as Table 1 shows.

**Table 1:** Foregone revenue from missing profits and hidden wealth in Malawi

**Missing Profits**	Corporate Income Tax as % of GDP in 2019	Foregone revenue as % GDP		Government revenue as % GDP in 2019	Foregone revenue as % of government revenue
	2.3	0.23		12.45	1.85
**Hidden Wealth**	Offshore wealth % GDP in 2007	Assuming 5% return on this wealth % GDP in 2007	Income tax foregone % GDP in 2007	Government revenue as % GDP in 2007	Foregone revenue as % of government revenue
	6.7	0.34	0.12	8.97	1.31

To calculate the foregone tax revenue from offshore wealth, we use the hidden wealth estimates for Malawi as of 2007 [69]. As explained earlier, the authors use this period because it is before the widespread use of shell companies. We assume a 5% return on offshore wealth and apply the upper tax bracket in Malawi of 35% on personal income. The resulting foregone tax revenue from hidden wealth is 1.31% of government revenue, as Table 1 shows.

**Fig. 1:**
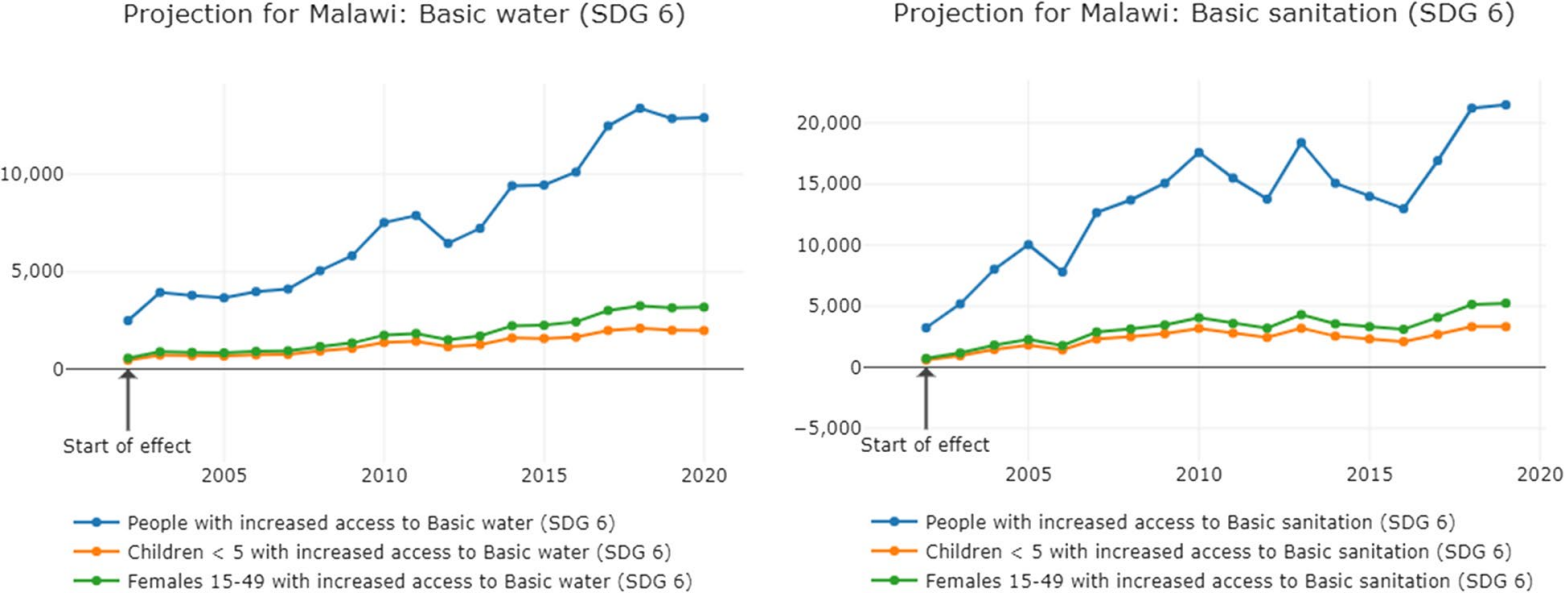
Increased coverage of SDG 6 equivalent to the government revenue losses from tax abuse in Malawi

**Fig. 2:**
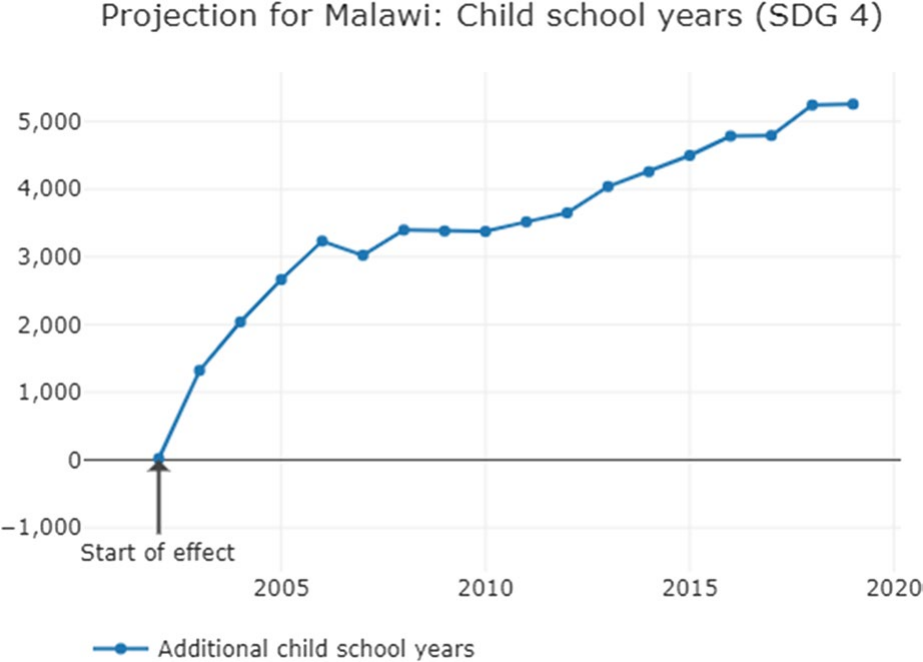
Increased coverage of SDG 4 equivalent to the government revenue losses from tax abuse in Malawi

**Fig. 3:**
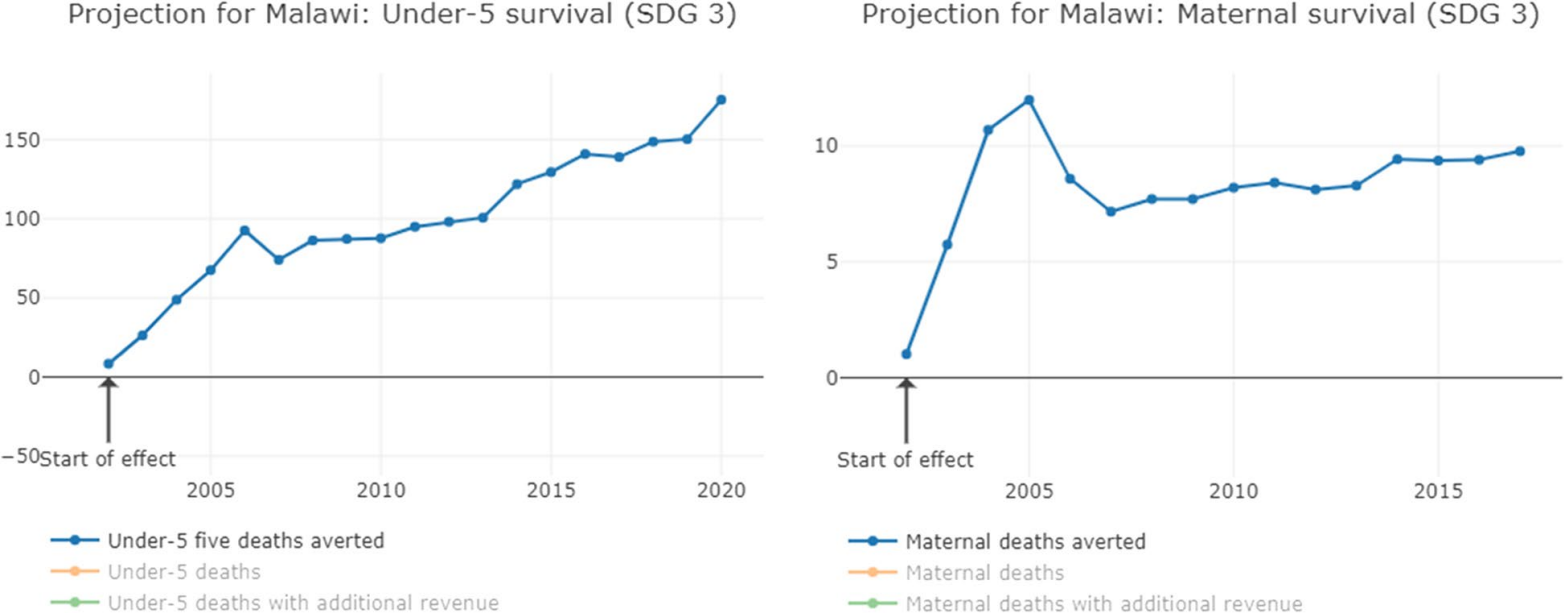
Increased coverage of SDG 3 equivalent to the government revenue losses from tax abuse in Malawi

In total, tax abuse results in a loss of 3.16% of government revenue in Malawi. Given that these estimates are based on indirect methodology, this is within the ballpark of revenue loss that O’Hare et al. identified in their study for Malawi (the absolute figure of US$51.31 million in 2010 USD expressed as government revenue in 2017 is 5.79%) which used the data set from the State of Tax Justice 2020 published by the Global Alliance for Tax Justice, Public Services International and the Tax Justice Network [31, 67]. These losses take place over decades, but we have point in time estimates for 2019 for missing profits and for 2007 for offshore wealth, expressed as percentages of government revenue for each year. We input the combined losses, which is 3.16% of government revenue, into the GRADE online visualisation across the maximum time possible, as it takes time for improved revenue to improve governance and SDG progress. We present additional numbers each year of people, females and/or children who would access their rights after the increases have plateaued. The variation in numbers is due to changes in the government revenue and governance indicators for each year, see Figs. 1, 2 and 3.

Additional government revenue, equivalent to corporate tax avoidance and evasion, would lead to 12,000 additional people, including children, accessing basic drinking water, and almost 20,000 accessing basic sanitation each year. Each year, an additional 5000 children would attend school, 150 additional children would survive and 10 more mothers would survive childbirth.

## Discussion

### Principal findings

If cross-border tax abuse were curtailed, the equivalent increase in government revenue in one country, Malawi, would be associated with 12,000 and 20,000 people having access to basic water and sanitation respectively each year. Each year, an additional 5000 children would attend school, 150 additional children would survive and 10 mothers would survive childbirth. There is an opportunity for the international community to contribute to progress towards the SDGs in Malawi by curbing global tax abuse. Pending the necessary reforms to the international tax system, the Malawian and other governments must take domestic action to mitigate the effect of the gap in global economic governance on children.

As explained in the background, countries have cross-border responsibilities for child rights in other countries, are duty bearers, and are required to act in terms of international cooperation to ensure all children have their rights fulfilled. In view of these obligations and the finding that there would be faster SDG progress if tax abuse was reduced, countries which facilitate tax abuse and governments of lower-income countries need to review their domestic policies to ensure all children have their rights met and greater progress is made towards all SDGs. We discuss recommended international and domestic action below.

### The way forward

#### International

The framework for taxing the profits of international business has been in place for 100 years. Decolonisation was accompanied by the movement of wealth and assets offshore, and former colonial powers strategically supported dependent states to set up low or zero tax regimes with little regulation for non-residents, both companies and individuals [82, 83]. During the mid-twentieth century, efforts by the United Nations to work on reforming international tax and transnational corporations faltered, and the forum shifted to less inclusive spaces, primarily the OECD [84].

For tax purposes, a multinational company earns profit as a single unit, but each subsidiary of the same parent company is treated as independent. The taxable profit is calculated using complex international treaties and domestic tax laws, including subsidiaries in corporate tax havens and secrecy jurisdictions, resulting in an immensely complex system that burdens revenue authorities and makes it possible for individuals to shift wealth and assets far away from where they reside and for companies to shift profits from where their actual economic activity happens to lower or no-tax jurisdictions. There is also a lack of clarity about allocating taxing rights on profits from cross-border corporations between the countries where these actual activities occur and where the company is headquartered and has subsidiaries [18]; and opacity in tax havens makes it possible for individuals to hide wealth and assets. Lower-income countries tend to lose more tax revenue relative to their total tax revenue due to corporate profit shifting; Africa, followed by Latin America, is disproportionately affected globally [85].

Yet some upper-middle and high-income (higher-income) countries and actors actively or passively facilitate these illicit financial flows [32, 86]. The State of Tax Justice 2020 estimates that 98% of the vulnerabilities to global abuses—both for corporate profit shifting and individual tax evasion on offshore wealth—are attributable to higher-income countries [67]. The Corporate Tax Haven Index shows the “UK’s dominant responsibility for corporate tax avoidance risks and the colonial roots of many exploitative double tax treaties” [87]. Indeed, the rules and regulations of the UK, along with Luxembourg, the Netherlands and Switzerland, and the network of tax treaties they have, including ones that predate the independence of African nations, create the vulnerabilities that enable more than half of global tax abuse [30]. Further, international listings of tax havens, such as the OECD’s ranking of peer reviews or the European Union’s list of non-cooperative jurisdictions, typically ignore the role their members and powerful states play in enabling financial secrecy, betraying the privilege and bias of the current global economic system [88].

Governments complicit in enabling tax abuse are duty bearers for the ensuing human and child rights abuses through eroding government revenue. Governments, especially development partners, are obliged to take urgent action, including steps to improve tax transparency and to review double tax agreements which may impede SDG progress in lower-income countries. As a foundation for reform, all governments must support negotiations within the auspices of the UN for a tax convention and global body to monitor taxing rights. Indeed, curtailing the flow of taxable profits from the countries where actual activities occurred to low-tax jurisdictions is an explicit aim of the wealthiest nations through the OECD/G20 BEPS project. The OECD, a successor to the imperial League of Nations, rather than the United Nations in setting the international tax rules to govern the practices of multinational corporations with their global operations [89]. However, given its mandate to represent the interests of its 38 member countries only, it is imperative to shift decision-making to a global, democratic forum in the UN with 193 member states. For example, in October 2021, the OECD/G20 Inclusive Framework released a statement on the “two-pillar solution” to address challenges arising from the digitalisation of the economy. It is a historic renegotiation of the current international tax rules and 137 jurisdictions endorsed the statement although there is presently no agreement in place to implement the first pillar [90]. And even though it is a historic renegotiation of the current international tax rules to ensure multinationals pay their fair share of tax, “both pillars directly and unambiguously privilege the global North in the allocation of taxing rights” [91]. In terms of the actual decision-making process “many developing countries have expressed concerns that the tax deal would only deepen inequalities between countries, and that the negotiation process was not inclusive and disregarded the reservations expressed by the Global South”; Kenya, Nigeria, Pakistan and Sri Lanka did not agree to the deal, and Malawi, and more than half of African jurisdictions, is not in the inclusive framework set up by the OECD to negotiate the agreement [92]. Further, there are multiple governance challenges with the framework, including pre-conditions to join the framework, alongside opacity and the limited influence of developing countries over negotiations and decision-making [91, 93, 94].

To effectively negotiate global tax matters and to monitor and evaluate the measures required to tackle tax abuse, it is increasingly recognised that a genuinely representative accountable body is necessary. The current international tax architecture is characterised by “a variety of different bodies, including the UN Tax Committee, the OECD and the Global Forum on Transparency and Exchange of Information for Tax Purposes, each with limited mandates and different configurations of membership, resulting in a somewhat haphazard set of overlapping international standards” [95]. Developing countries through the Group of 77 (G77) plus China have repeatedly, since 2011, called for an inclusive intergovernmental forum [96]. The report of the High Level Panel of Illicit Financial Flows from Africa in 2015 suggested that the UN processes and frameworks would be the ideal forum for international tax coordination (finding 15). The African Group advocated for a UN Convention on Tax in 2019, and in 2020, the UN Secretary-General identified a Convention on Tax as an essential option to consider [97]. Its absence is a massive gap in global economic governance and negatively impacts economic and social human rights [98].

In 2021, as a follow-up to the Mbeki Report, the UN High-Level Panel on International Financial Accountability, Transparency and Integrity (FACTI) [99] laid out steps for SDG progress: the FACTI panel states that all countries must grapple with illicit financial flows. One of their key recommendations is creating a fair mechanism to arbitrate international tax disputes under a UN Tax Convention [99]. The FACTI panel report and these recommendations were welcomed by the Africa Group of Finance Ministers at the UN Forum on Financing for Development, where Vice President of Malawi Saulos Chilima, speaking on behalf of the Africa Group in early 2022, said.

Therefore﻿, the African Group firmly believe in the urgent need to establish a universal UN intergovernmental tax body and negotiate a UN Tax Convention to comprehensively address tax havens, tax abuse by multinational corporations and other illicit financial flows through a genuinely universal, intergovernmental process at the UN, with broad rights holders’ participation […]

We believe that, if implemented, have the potential to significantly reduce existing structures that make it impossible for countries to generate and retain a sizeable chunk of their resources [100].

At the end of 2022, Nigeria, on behalf of the African Group, put forward a resolution 77/244 on the ‘Promotion of inclusive and effective tax cooperation at the United Nations’. This was adopted by the UN General Assembly with unanimous consensus and marks the beginning of intergovernmental discussions on international tax cooperation, with “the possibility of developing an international tax cooperation framework or instrument that is developed and agreed upon through a United Nations intergovernmental process” [101]. At the Second Committee stage, efforts—that ultimately proved unsuccessful—were made to delete this language from the draft resolution by the US delegation to the UN [102]. These efforts were supported by some of the countries that most enable tax abuse, including the Netherlands, Luxembourg, the UK, and Switzerland [103]. A UN framework on tax could establish an inclusive intergovernmental global tax body so that all countries can participate and ensure linkages to development, human rights and environmental protection, as also outlined in the SDGs [95]. It could also introduce a new tax system to replace the current failing transfer pricing system so that companies are taxed on their group profits and based on an agreed formula with a minimum effective tax rate so that tax rights are allocated across countries where a multinationals operates [87, 92]. A UN tax convention could contain and monitor key tax transparency measures required to tackle tax abuse; these are commonly known by the acronym ABC: Automatic exchange of information, Beneficial ownership registration, and public Country-by-country reporting [104, 105], incorporated into the UN FACTI panel recommendations [99], building on the Mbeki Report [23]. In mid 2023, the Secretary General of the UN published the *Tax Report 2023* [106] based on analysis of the current international tax system and submissions from different stakeholders. The report finds that enhancing the UN’s role “in tax-norm shaping and rule-setting […] appears the most viable path for making international tax cooperation fully inclusive and more effective” [106] and three options are presented for UN members’ consideration. At the UN General Assembly in September 2023, consensus was emerging around the second option, for a framework convention on international tax cooperation, ahead of a likely vote on a specific resolution submitted by the African Group in late 2023 [107]. However, the US, UK and the EU and dependencies were notably silent at the General Assembly, and EU finance ministers subsequently released a statement giving support only to option three which is a non-binding reform [108].

#### Domestic

Until the establishment of an intergovernmental body to monitor taxing rights and an international tax cooperation framework, the governments of Malawi and lower-income countries need to continue to mitigate the impact of gaps in global governance and protect themselves from illicit financial flows, including corporate profit shifting and tax evasion through undeclared offshore wealth, and improve the effectiveness of the use of revenue domestically. Malawi’s Domestic Revenue Mobilisation Strategy (DRMS) 2021–2026 and the National Anti-Corruption Strategy II, 2019–2024, are vital parts of Malawi’s blueprint for tackling illicit financial flows, ensuring taxes are collected and assets are recovered, showing the Malawian government’s “obligation to improve service delivery and the quality of life of all Malawians”, as it states in the DRMS [52, 109]. Here we draw out and discuss the domestic recommendations from the FACTI panel [99]. The panel summarises the current selected mechanisms for financial accountability, transparency, and integrity and puts forward 14 recommendations in three main areas, values (1–5), policies (6–10) and institutions (11–14). We identify seven as having particular relevance for domestic action, as Table 2 shows.

**Table 2:** The recommendations of the FACTI panel and the current policies in Malawi

**Recommendation**		**Domestic context**
Corruption	From the United Nations High-Level Panel on International Financial Accountability, Transparency and Integrity (The FACTI panel) FACTI Panel Recommendation ‘Accountability’ (1A): Enhancing the effective implementation of the United Nations Convention Against Corruption (UNCAC) is critical for improved accountability. All countries should enact legislation providing for the widest possible range of legal tools to pursue cross-border financial crimes.	Malawi signed and ratified UNCAC, the only legally binding global anti-corruption convention, in 2004 and 2007, respectively. It also ratified the African Union (AU) Convention on Preventing and Combatting Corruption in 2007 and the Southern African Development Community (SADC) Protocol against Corruption. Further improvements to effectively implement the global and continental conventions, as well as the SADC protocol to specifically tackle cross-border financial crimes include: • Comprehensively reviewing the multi-layered legal framework because of overlaps and discrepancies arising from the conflict between some provisions from the colonial period and modern legislation. This includes reviewing the Penal Code, the Corrupt Practices Act, the Public Finance Management Act, the Financial Crimes Act, Public Procurement and Disposal of Public Assets Act, Criminal Procedure and Evidence Code, the Police Act, the Dangerous Drugs Act, the Customs and Excise Act, and the National Parks and Wildlife Act. • Specifically, the definition of theft in the Penal Code is inadequate because it is based on the “larceny notion of taking away a portable thing: It is unfit for offences involving interference with rights over incorporeal property, such as balances in bank accounts, electronic transfer of funds and votes entered into the IFMIS [Integrated Financial Management Information System]” [110]. • Development of sentencing guidelines for financial crime and corruption offences (ibid). • The Courts Act Amendment Bill of 2022 was passed in Parliament on 2 August 2022, and this Bill, if assented to, will see the establishment of the Financial Crimes Division in the High Court to handle corruption and financial crimes cases [111]. • On 28 July 2022, Parliament passed the Corrupt Practices Act Amendment Bill and this Bill, this empowers the Anti-Corruption Bureau to prosecute a matter without obtaining consent from the Director of Public Prosecutions Office, thus speeding up the handling of corruption cases [112].
Transparency	From FACTI Panel Recommendation ‘Transparency’ (3A): Secrecy flourishes because of inconsistent and ineffective beneficial ownership information regimes. Public registry of all beneficial ownership of all legal vehicles.	Identifying the ‘beneficial owners’, or the natural persons who ultimately own, control or benefit from legal vehicles, including companies, partnerships, trusts and foundations, is central to financial transparency and can, for example, “reveal that apparently legitimate and unrelated companies and trusts are in fact implicated in global financial crime or tax abuse scheme” [99]. The best practice requires all owners (without a control or ownership threshold) to be disclosed, including identifying information, for all legal vehicles and for this to be updated with sanctions for non-compliance and systems to verify submitted information [113]. All the information should be housed in a central and public register. • The second National Anti-Corruption Strategy (NACS II) (2019-2024) identifies beneficial ownership as part of its objectives in addressing all weaknesses in the legal framework to align it with international best practices in the fight against corruption. • Malawi should introduce a public registry for the disclosure of beneficial owners of companies, partnerships, and trusts, managed by the Department of the Registrar General. This should require all beneficial owners to be disclosed without a threshold, as is the case in Botswana [114], and made publicly available free of charge online, like in Nigeria. • In December 2022, the Companies (Beneficial Ownership) Regulations were gazetted, requiring companies to disclose the beneficial owners with a 5% threshold; according to Section 11(1), “Any beneficial ownership information shall be treated as public information and may be accessible to the public”. • This development, built of Malawi’s commitments to beneficial ownership transparency in the extractive industries and in public procurement relating to the expenditure of IMF financing in the response to Covid-19 [115]. • Asset declaration assists in preventing and fighting corruption and money laundering as well as in upholding the public trust in the public service. Publicly elected officials and officers of a certain grade and profession in the public service under sections 14 and 15 of the Public Officers (Declarations of Assets, Liabilities and Business Interests) Act 2014 are supposed to declare inter alia their ownership in any assets and business interests to the Office of the Director of Public Declarations [116]. Accountants, procurement officers and lawyers working in certain offices are included in the list of officers supposed to make these declarations. Section 17 of the same Act allows the public to access declarations, which can be made public upon request to the Director. However, declarations are treated as confidential and a list of politically exposed persons should be published [54].
	From FACTI Panel Recommendation ‘Transparency’ (3B): Public country-by-country reporting of all private multinational companies	Multinational companies should publish a country-by-country report that includes disaggregated data on the corporate group’s activity, including revenue, profit and loss, taxes paid, and the number of employees. This makes it possible to better target audits and investigations of tax avoidance and evasion. In the absence of global public country by country reporting standards, a second-best scenario is a requirement for the local filing of country-by-country reports, which means that “authorities of all countries where a multinational has operations can access reports in cases where these reports cannot be obtained through automatic exchanges, regardless of the reason” [117]. Malawi should introduce this requirement, especially since Malawi is not a signatory to the G20/OECD Common Reporting Standard Multilateral Competent Authority Agreement, which is required to automatically exchange country-by-country reports [118]. Even if Malawi were a signatory, there are prerequisites placed on tax authorities for exchanges and limits placed on the use of reports that are exchanged [119].
	From FACTI Panel Recommendation ‘Transparency’ (3C): Building on existing voluntary efforts, all countries should strengthen public procurement and contracting transparency	Malawi’s Public Procurement and Disposal of Public Assets Act of 2017 lays the groundwork for open contracting and the NACS II makes commitments to reduce public procurement corruption. The government through the Public Procurement and Disposal of Assets Authority (PPDA) is implementing open contracting initiatives, which will promote the principles of transparency enshrined under sections 30 and 31 the Act [120]; however, procuring entities are not fully complying with disclosure requirements and the online open data portal is not yet fully operational [121]. The open contracting initiative is in pursuance of section 57(2) of the Public Procurement and Disposal of Public Assets Act which stipulates that a procuring entity is obligated to disclose information that may affect a procurement process to the party who has any interest in the said process [122]. The PPDA has launched an e-services platform as a means of making procurement services more efficient. This is in line with the mandate of the Public Procurement and Disposal of Assets Authority provided under section 5 of the primary legislation that states that the Authority shall be responsible for developing and enhancing the efficiency of procurement and disposal of assets processes.
Fairness in taxation	From FACTI Panel Recommendation ‘Fairness’ (4A): Taxpayers, especially multinational corporations, should pay their fair share of taxes. The UN Tax Convention should provide for effective capital gains taxation. Taxation must be equitably applied on services delivered digitally.	In Malawi’s Domestic Revenue Mobilisation Strategy (DRMS) 2021-2026, the government includes a number of activities that feed into enabling the government to pursue cross-border financial crimes and tax abuse [52]. This includes revisiting old and disadvantageous tax treaties and countering tax abuse in line with the BEPS action plans, alongside strengthening audit capacity, with specific focus on banking, insurance, telecommunications, mining, and construction. Malawi has a dedicated office for large taxpayers and transfer pricing legislation, which assist in the taxation of multinational corporations. Yet the DRMS does not specifically mention illicit financial flows or trade misinvoicing. The digitalised economy poses new challenges for taxation and in an ideal situation, to tax digital services provided by multinational companies, multinational corporations should be taxed with a formulary apportionment approach based on group global profit. Yet in the absence of this and Malawi could carry out a cost-benefit analysis and follow Kenya, Nigeria, and Tanzania by applying a unilateral (direct) tax on non-resident digital service providers. Malawi should review all double tax treaties in force, especially one signed before Malawi’s independence, such as with the United Kingdom (1955), Switzerland (1961) and France (1963), to ensure provisions favour greater retention of taxing rights of source countries compared to the residence country of investors [123]. Particular attention should also be paid to current tax treaties under negotiation or those that are already initialled, such as one initialled with Mauritius in 2017.
Enablers	From FACTI Panel Recommendation ‘Enablers’ (6): Developing countries are systemically disadvantaged in the current international tax architecture. Governments should develop and agree global standards/guidelines for financial, legal, accounting, and other relevant professionals, with input of the international community	Enablers refer to a set of professionals, including lawyers, accountants, and bankers, that may support individuals and companies in tax abuse. The FACTI Panel observes the abuse of legal privilege to assist in money laundering and tax abuse, that self-regulation by enablers, by their institutions and by law associations is insufficient and unreliable, and despite the social costs, many governments have not set standards or do not enforce these standards in the public interest. • The most recent mutual evaluation review of Malawi conducted by the Eastern and Southern Africa Anti-Money Laundering Group (ESAAMLG) in 2018 to assess the level of Malawi’s implementation of the Financial Action Task Force (FATF) recommendations on anti-money laundering (AML) and counter-terrorist financing (CFT) includes an evaluation of designated non-financial businesses and professions (DNFBPs). This includes lawyers and accountants. The report states that in Malawi “the DNFBP sector is a fairly small player and therefore it has a minimal integration into the global financial system”. In addition, “The lawyers, accountants and real estate agents demonstrated a good application of AML/CFT [Anti-Money Laundering/Combatting the Financing of Terrorist] [… but] with the exception of banks, filing of STRs [Suspicious Transaction Report] by the other FIs [Financial Institutions] and DNFBP sector is low”. There is also room for improvement in understanding among law firms about the risks of in relation to beneficial ownership information of companies, trusts and other legal vehicle, and accounting firms along with other DNFBPs, except law firms, were recommended to improve enhanced and on-going due diligence [124]. • **Supervision. **Under sections 4 and 5 of the Financial Crimes Act, the Financial Intelligence Authority can receive and request reports from DNFBPs and instruct them to take steps to facilitate an investigation anticipated by the Authority. The said Act under section 5 also permits the Authority to issue guidelines to supervisory bodies for the better carrying out of its functions. It can also delegate its powers to supervisory bodies that regulate certain professions. In Malawi, lawyers are regulated by the Malawi Law Society whilst accountants are regulated by the Malawi Accountants Board and supervised by Institute of Chartered Accountants as mandated by the Public Accountants and Auditors’ Act 2013. A risk-based supervision system was not in place at the time of assessment for DNFBPS, and only for supervision of financial institutions. • **Sanctions. **No sanctions had been applied for violation of AML/CFT requirements by DNFBPs according to the ESAAMLG: “no sanctions have been given to DNFBPs in the period under review while limited remedial actions have been issued to DNFBPs that have been inspected. Therefore, it is difficult to determine if in practice, the sanctions are dissuasive, proportionate, and effective in respect of the DNFBPs”. That said, recommendation 28 on regulation and supervision of DNFBPs was assessed as met [124]. Section 23 of the Financial Crimes Act is the law that instructs financial institutions and DNFBPs to report suspicious transactions to the Financial Intelligence Authority. Sanctions for non-compliance with the Act are provided in section 24 and according to the Money Laundering and Terrorist Financing National Risk Assessment report, prepared by Malawi’s Financial Intelligence Authority, no criminal sanctions have been imposed on lawyers [53]. However, three lawyers suspected to be involved in the Cashgate scandal were charged under the old Money Laundering Act and their matters are now in court. The lawyers were not sanctioned by the Malawi Law Society for their alleged involvement in the scandal. • **Reporting. **A further measure would be to introduce rules to collect intelligence from tax advisors and taxpayers about complex and risky tax avoidance practices. Such as, mandatory rules requiring tax advisors and taxpayers to report to the Malawi Revenue Authority 1) any tax avoidance schemes marketed/sold or used, and 2) on uncertain tax positions for which reserves have been created in annual corporate accounts [125]. At present, lawyers are expected to report to the Financial Intelligence Authority any transaction that surpasses 1 million MWK.
Civil society actors, whistle-blowers, and journalists	From FACTI Panel Recommendation ‘Non-State Actors’ (7A): States should consider incorporating minimum standards of protection for human right defenders, anticorruption advocates, investigative journalists and whistle-blowers in a legally binding international instrument	**Whistle-blowers: **The legal framework to protect whistle-blowers is inadequate, “due to bureaucratic culture, lack of incentives and feelings of insecurity by would-be whistle-blowers” [110]. Development of a comprehensive legal framework is the first step, especially ensuring protection alongside incentivising whistle-blowing through rewards and providing access to remedy and compensation for victimised whistle-blowers. However, the Platform to Protect Whistle-blowers in Africa indicates in their assessment of Malawi that compromised integrity of some officials working in law enforcement agencies disincentivises would-be whistle-blowers and increases the risks for those who do blow the whistle [126]. In the absence of a comprehensive legal framework, the following existing laws afford some protection to whistle-blowers, including: • Section 51A of the Corrupt Practices Act provides protection for whistle-blowers by stating that their identity will not be disclosed in criminal or civil proceedings unless the court believes after full inquiry into the case that the whistle-blower intentionally provided information that he knew to be false or the court believes that it is in the interests of justice that the whistle-blower’s identity is disclosed [127]. • Section 20 of the Public Officers (Declarations of Assets, Liabilities, Business Interests) Act provides protection for whistle-blowers by stating that their information shall not be submitted as evidence in a court proceedings and no person shall be obliged to disclose the identity of the whistle-blower [116]. The provision, however, gives an exception to that protection by stating that if the court or the Director after inquiry into the case determines that the whistle-blower deliberately gave false information to the Director, the immediate disclosure of the identity of the whistle-blower will be required. That said, section 21 of the said Act provides protection for the whistle-blower by stating that anyone who reveals the identity of the whistle-blower commits an offence and shall on conviction, face the penalty of MWK500,000 and two years’ imprisonment. • Section 50 of the Access to Information Act states that a person will not be penalised for disclosing information regarding corruption that they obtained in confidence in the discharge of their professional duty, if the disclosure is in the interests of the public [128]. • In the Corruption Prevention Policy for the Public Procurement and Disposal of Assets Authority, it states that where an employee in good faith, reports inter alia suspected corruption, the Director shall take reasonable steps to protect them from victimisation [129]. • There is also an informant scheme introduced in some agencies, including by the Malawi Revenue Authority with both an in-house informant scheme and Deloitte Tip-Offs Anonymous for members of public to inform the Malawi Revenue Authority or the police of suspected cases of tax evasion and get a reward in return [130]. This act by the public is stated to enable the Government to collect more revenue and as a consequence provide services to the public such as health, education and justice [131]. **Human rights defenders and journalists** face intimidation. Reporters Without Borders in their assessment of Malawi considers that political influence over the media restricts journalistic freedom, and journalists are subjected to threats and online harassment [132]. As recently as April 2022, an investigative journalist was detained and questioned by the police, in an attempt to force him to disclose sources in relation to reporting about alleged corruption and financial crime [133, 134]. In its assessment of Malawi, Freedom House indicates that the vague wording in the 2016 cybersecurity law (Section 87, Electronic Transaction and Cyber Security Act) on offensive communication could threaten online freedom of speech and press [135] alongside older provisions in the penal code and the Protected Flag, Emblems and Names Act that stifle free speech online and offline [136]. **Access to information: **Section 5 of the Access to Information Act of 2016 permits people to request information from public and private bodies in exercise of their rights [130]. However, under sections 30 to 36, there are among others, limitations for the disclosure of that information; for instance, the institution may refuse to disclose the information if it is legally privileged, or its disclosure would threaten national security. Regulations were enacted in 2021 [137]. **Data protection: **Broadly, and important for all citizens and not just civil society actors, whistle-blowers and journalists, Malawi requires a robust data protection framework, especially given the escalation in personal data collection in the last five years. The introduction of the national digital ID programme in 2017 and mandatory SIM card registration in 2018 necessitate urgent finalisation and passage of the draft Data Protection Bill of 2021 [138].
Automatic Exchange of Information	From FACTI Panel Recommendation ‘International Cooperation’ (8): End asymmetries and promote free exchange of information to combat illicit financial flows	The automatic exchange of information between jurisdictions is intended to provide a jurisdiction with data on of all the financial activity abroad of its residents to prevent residents concealing the true value of financial activity and enable law enforcement agencies to ascertain that residents are paying the correct amount of tax and investigate tax evasion and other crimes. The current framework promoted by the OECD and G20 includes the Common Reporting Standard and the Multilateral Competent Authority Agreement to implement the standards alongside the OECD Convention on Mutual Administrative Assistance in Tax Matters for the exchange of information on request. Yet there is “defacto exclusion of developing countries” based on the design and prerequisites to receive information as well as restrictions on data usage by law enforcement agencies [139]. Notwithstanding fundamental challenges with the current international framework for the exchange of information on request and the automatic exchange of information, Malawi should become signatory given these are the primary tools for the exchange of information on request multilateral automatic exchange of information and could help the Malawian government address tax-motivated illicit financial flows [140].
National systems and coordination	From FACTI Panel Recommendation ‘Dynamism’ (9): Governments must dynamically adjust their national and international systems in response to new risks.	Nimble adaptation based on rapid technological developments is central to tackling illicit financial flows. In particular, the rise of virtual asset service providers, such as those providing cryptocurrencies, has widespread implications for anti-money laundering and efforts to stop tax abuse. Section 21 of the Financial Crimes Act instructs financial institutions to conduct money laundering and terrorist financing (ML/TF) risk assessments with regards to the products offered and delivery channels and to take counter measures commensurate the risk. At the time of the last assessment of Malawi by the Eastern and Southern Africa Anti-Money Laundering Group, Malawi had not identified and carried out an ML/TF risk assessment associated with the development of new products and new business practices, including new delivery mechanisms, and the use of new technologies for both new and existing products [124]. In the same year, in 2019, the Reserve Bank of Malawi issued a statement informing the public that cryptocurrencies are not a legal tender in Malawi and the Central Bank has no oversight responsibility over cryptocurrency trade [141]. More recently, in April 2022, Malawi’s Financial Intelligence Authority conducted meetings sensitising anti-money laundering stakeholders on virtual assets risks and in these meetings the participants were told to take part in the exercise as the risk assessment findings will help Government policymakers to determine an appropriate policy position to be taken on virtual assets and virtual asset service providers [142].
	From FACTI Panel Recommendation ‘National Governance’ (13): Governments should create robust and coordinated national governance mechanisms that efficiently reinforce financial integrity for sustainable development and publish national reviews evaluating their own performance.	Whole-of-government systems are needed for a holistic approach to ensure all types of illicit financial flows are covered and a range of expertise is applied [140]. The Money Laundering and Terrorist Financing National Risk Assessment Report (2018) explains that the National AML/CFT Committee, set up formally in September 2017, facilitates close cooperation and information sharing required for effective AML/ CFT in the country [53]. Before this, there was a committee that met on an ad hoc basis and was not formal. The current committee is co-chaired by the Secretary to the Treasury and the Director General of Financial Intelligence Authority; however, its powers and responsibilities are not sanctioned by any law, which means its decisions are not legally binding. The Risk Assessment also identifies room for improvement in coordination, including in asset forfeiture and between the Financial Intelligence Authority and other law enforcement agencies. Individual institutions, including the Reserve Bank of Malawi, the Financial Intelligence Authority, the Malawi Policy Service (Fiscal and Fraud Department) and the Malawi Revenue Authority, all have a mandate to investigate. The recent, ongoing, high profile joint UK-Malawi investigations [143, 144] into a British citizen of alleged billions of Malawi kwacha of illegally acquired wealth from Malawi primarily through alleged procurement fraud suggest that improved coordination, among other areas, is needed in investigation, and between agencies responsible for investigation and prosecution [145]. These investigations also point to the potential greater cooperation and information sharing between Malawi and other jurisdictions in investigation with the aim of prosecution and asset recovery. On tax evasion, there are several cases that the Malawi Revenue Authority have in the courts relating to transfer pricing. In total, about MWK100 billion in tax revenues is involved in these cases. However, the limited progress may be in part explained by challenges with specialisation to litigate such cases, which will likely be addressed partially by the establishment of a division in the High Court for financial crimes [146].

The recommendations of the FACTI panel and the current policies in Malawi are summarised in Table 2. and we consider these a whole-of-government approach to tackling illicit financial flows. Nevertheless, these are primarily technical and would be well served by political economy analysis to ensure effectiveness.

## Conclusion

More children would access their economic and social rights if there were actions by duty-bearers to close the vast gaps in global governance regarding tax. To illustrate this, we selected Malawi and the impact of revenue losses because of corporate and individual tax abuse on fundamental rights, which are also determinants of health.

We have discussed the responsibility of the international community, with the onus on those countries which create the most vulnerabilities to tax abuse—Luxembourg, the United Kingdom and dependencies, the Netherlands and Switzerland—and which attempted, albeit unsuccessfully, to thwart efforts by the African Group at the UN to begin intergovernmental discussions with the possibility of developing an international tax cooperation framework. Yet these nations have ratified the UNCRC and have obligations to children globally. We urge all countries to support efforts for international tax to be determined and decided through the UN. This is in line with the 16^th^ SDG, which aims to reduce all forms of illicit financial flows significantly. As per the recommendations of the FACTI panel, Malawi, and indeed all countries, can continue to mitigate the impact of the gaps in the international tax architecture that have emerged and been engineered over the last century to better secure the future of the children with their actions to tackle illicit financial flows domestically.

## Data Availability

All data is available on the GRADE website (https://www.st-andrews.ac.uk/~grade/doh/), the Missing Profits (https://missingprofits.world/), Hidden Wealth (https://gabriel-zucman.eu/hidden-wealth/), Who Owns the Wealth in Tax Havens? (http://gabriel-zucman.eu/offshore/) websites, and the Tax Justice Network’s website (https://iff.taxjustice.net/).

## Abbreviations

ABC: Automatic exchange of information, Beneficial ownership registration and public Country-by-country reporting
AML/CFT: Anti-money laundering and countering the financing of terrorism
BEPS: Base Erosion and Profit Shifting
DNFBPs: Designated non-financial businesses and professions
DRMS: Malawi’s Domestic Revenue Mobilisation Strategy
ESAAMLG: Eastern and Southern Africa Anti-Money Laundering Group
FACTI: United Nations High-Level Panel on International Financial Accountability, Transparency and Integrity
FATF: Financial Action Task Force
G20: Group of Twenty (a UN forum comprising 19 countries and the European Union)
G77: Group of Seventy-Seven (a UN forum comprising 134 developing countries)
GDP: Gross Domestic Product
GRADE: Government Revenue and Development Estimations project
ML/TF: Money laundering and terrorist financing
MWK: Malawian Kwacha
NACS II: The second National Anti-Corruption Strategy
OECD: Organisation for Economic Co-operation and Development
PPDA: Public Procurement and Disposal of Assets Authority
SADC: Southern African Development Community
SDGs: Sustainable Development Goals
UN: United Nations
UNCAC: United Nations Convention Against Corruption
UNCTAD: United Nations Conference on Trade and Development
UNCRC: United Nations Convention on the Rights of the Child
USD: United States Dollars
WDI: World Development Indicators
WGI: Worldwide Governance Indicators

## Footnotes

1 We use the term lower-income countries to include low-income and lower-middle income countries as defined by the World Bank. We use the term higher-income to include upper-middle-income and high-income countries as defined by the World Bank.
